# Nab-paclitaxel and gemcitabine or FOLFIRINOX as first-line treatment in patients with unresectable adenocarcinoma of the pancreas: does sequence matter?

**DOI:** 10.1186/s12885-018-5240-6

**Published:** 2019-01-08

**Authors:** Ursula M. Vogl, Haleh Andalibi, Alexander Klaus, Laurenz Vormittag, Wolfgang Schima, Bettina Heinrich, Alice Kafka, Thomas Winkler, Leopold Öhler

**Affiliations:** 1 Department of Internal Medicine II, Oncology, Barmherzige Schwestern Wien, Stumpergasse 13, 1060 Vienna, Austria; 2 Department of Surgery, Barmherzige Schwestern Wien, Vienna, Austria; 3 Department of Diagnostic and Interventional Radiology, Barmherzige Schwestern Krankenhaus Wien, Goettlicher Heiland Krankenhaus and Sankt Josef Krankenhaus, Vienna, Austria; 4 Department of Internal Medicine I, Oncology, Sankt Josef Krankenhaus, Vienna, Austria

**Keywords:** Pancreatic cancer, FOLFIRINOX, Nab-paclitaxel, Gemcitabine

## Abstract

**Background:**

Locally advanced or metastatic adenocarcinoma of the pancreas remains - despite the implementation of new chemotherapy protocols - a disease with short overall survival (OS).

**Methods:**

Eighty-three patients were treated with locally advanced or metastatic adenocarcinoma of the pancreas with either FOLFIRINOX or nab-Paclitxel and Gemcitabine (nabPGem) as first- or second line therapy. We analysed the outcome for OS and progression-free survival (PFS) in terms of treatment regimen and sequence.

**Results:**

The majority of patients presented in good performance status (PS) with a median age of 68 years. Fourty-two patients received FOLFIRINOX as first-line therapy, 41 patients were treated with nabPGem as first line therapy. Forty-eight patients received both treatments. The OS of all 83 patients was 12.6 months (95% CI: 10.7–14.6), resulting in a 1-year OS of 54%. Forty-eight patients received FOLFIRINOX followed by nabPGem or vice versa. There was no significant difference in OS or PFS for either of the two sequences (*p* = 0.9). The OS for FOLFIRINOX followed by nabPGem or nabPGem followed by FOLFIRINOX was 13.7 months (95% CI: 12.6–14.7) and 13.8 months (95% CI: 8.6–19), respectively.

**Conclusions:**

The sequence FOLFIRINOX followed by nab-Paclitaxel and Gemcitabine or vice versa lead to an equal OS outcome.

## Background

Metastatic or locally advanced inoperable adenocarcinoma of the pancreas has a dismal prognosis and is projected to be the second-most lethal cancer type by the year 2030 [1]. Despite intensive research in this field, none of the novel antineoplastic agents, such as checkpoint inhibitors or targeted agents, have shown any striking effect in in larger randomized trials in the overall population [2–8]. Some promising data have been presented in early phase I and II trials with PARP (poly ADP ribose polymerase) inhibitors and immunotherapy for the rare patients with BRCA (breast cancer type 1 susceptibility protein) mutations and MSI (microsatellite instability) high or dMMR (deficient mismatch repair) tumours [9–12]. Only a small number of cytotoxic drugs and one targeted agent (Erlotinib) have improved the outcome of this devastating disease [13]. Even when detected early, operable pancreatic cancer relapses in more than half of patients [14].

In the late 1990s, gemcitabine was implemented as the standard of care in first-line treatment of metastatic pancreatic cancer, demonstrating an overall survival (OS) benefit over 5-fluouracil (5-FU) [15]. The genomic structure of metastatic pancreatic cancer is very complex. To date, no significant genomic alteration that is targetable with drugs has been described. Most tumours carry rat sarcoma (RAS) or tumour protein (TP)53 mutations, which make pancreatic cancer unsuitable for precision medicine with targeted agents [16]. In 2011, the triple chemotherapy 5-FU, leucovorin, irinotecan and oxaliplatin (FOLFIRINOX) significantly improved response rates and OS compared to gemcitabine as a first-line treatment in the PRODIGE/ACCORD trial [17]. The high toxicity of this regimen, however, limits its availability to patients with an Eastern Cooperative Oncology Group (ECOG) status of 0 or 1. ECOG 2 patients were not included in the trial. The superior objective response rate (ORR) makes FOLFIRINOX the actual treatment of choice for borderline resectable tumours in a neoadjuvant treatment concept. In 2013, the MPACT phase III trial, which included over 800 patients, showed a significant OS benefit with the combination of nanomolecular albumin-bound (nab)-paclitaxel and gemcitabine (nabPGem) over gemcitabine monotherapy, with acceptable toxicity [17, 18]. This new nab-paclitaxel offers a higher concentration of the drug in the tumour stroma and endothelial cells and increases the potency of gemcitabine. The overall toxicity is lower than in FOLFIRINOX, especially haematotoxicity and rates of neutropenic fever. In both large randomized trials, approximately 40 to 50% of the patients received second-line treatment, mostly 5-FU- or oxaliplatin-based [18]. At the time of trial recruitment (Prodige, MPACT), new drugs such as nal-irinotecan (nal-IRI) had not been offered to patients in later lines. Nal-IRI in combination with 5-FU/leucovorin had shown a benefit in gemcitabine-pretreated patients in the NAPOLI trial [19]. None of the trials, however, described patients receiving chemotherapy beyond second-line treatment.

Current guidelines recommend nabPGem or FOLFIRINOX as first-line treatment, followed by nal-IRI depending on the patient’s performance status (PS). There is no randomized trial - and therefore only limited retrospective data - directly comparing these two first-line regimens, thereby leaving the choice up to the treating physician [20, 21].

Patients with locally advanced, unresectable disease are underrepresented in clinical trials for the treatment of metastatic disease. Therefore, there is a lack of data concerning the management of this group of patients. Current guidelines from the European Society of Medical Oncology (ESMO) recommend gemcitabine monotherapy as the standard of care for these patients or chemoradiation with capecitabine. The National Comprehensive Cancer Network (NCCN) guidelines extrapolated data from patients with metastatic pancreatic cancer and recommended the use of nabPGem or FOLFIRINOX with a category of 2A [22]. After an induction period of 3 months, chemoradiation can be discussed for better local control.

In this retrospective analysis, we report the outcome of patients with locally advanced, inoperable or metastatic disease who received palliative chemotherapy with either FOLFIRINOX or nabPGem as first-line treatment in our institution over the past 7 years.

## Methods

Between 2012 and 2018, 83 patients with locally advanced or metastatic pancreatic adenocarcinoma were treated at our institution. Nab-paclitaxel was licensed in Austria in 2013, and thus early patients in this retrospective analysis were treated off-label based on available phase II data [18]. Gemcitabine was administered with 1000 mg/m^2^ after application of nab-paclitaxel (125 mg/m^2^) on days 1, 8 and 15 every 28 days. Patients with ECOG ≥2 received nabPGem at the same dose biweekly. FOLFIRINOX was given every 2 weeks as described [23]. Modified FOLFIRINOX (mFOLFIRINOX) was administered at a reduced dose (80% dose of all chemotherapeutic agents) in 22% of patients. OS was measured from the start of first palliative chemotherapy until death. The response rates were evaluated using Response Evaluation Criteria in Solid Tumors (RECIST 1.1) criteria [24]. Adverse events were evaluated and graded through review of chart documentation according to the Common Terminology Criteria for Adverse Events (CTCAE version 4.0). CA 19–9 was considered elevated when equal to or greater than 37 U/ml. A significant CA 19–9 response was defined as a decrease greater than 50% from an elevated baseline. The median PFS and OS were determined using the Kaplan-Meier method. Statistical analysis was performed using SPSS for Windows v23. Statistical significance was indicated by *p* < 0.05 using the log rank and Breslow test. Statistical consultancy during data analysis was provided by “Clinical Trials Group Austria”.

## Results

A total of 83 patients were included in our retrospective analysis. The patients’ baseline characteristics are shown in Table 1. The median age was 68 years (range 31–93), and 57% were male. More than half of the patients presented with ECOG 0, but 12% presented with ECOG 2. At the time of initiation of systemic chemotherapy, 21 patients (25%) had locally advanced, inoperable disease. The majority of the metastatic disease sites were in the liver (65%), with 23% peritoneal carcinomatosis and 24% lung at the time of diagnosis. Nineteen percent of the patients had more than one metastatic site at the time of initiation of palliative chemotherapy. Baseline levels of CA 19–9 were significantly elevated in 83% of all patients with a median of 1363 U/ml (range 0–328,209). A small proportion of patients had a negative CA 19–9 at baseline. Ten percent of patients received prior adjuvant chemotherapy.

**Table 1:** Patient demographics and baseline characteristics (*n* = 83)

Patient characteristics	*N*	%
Median age (range)	68	(31–93)
Sex		
- male	47	57
- female	36	43
ECOG		
- 0	49	59
- 1	24	29
- 2	10	12
Stage		
- IV	62	75
- III inoperable	21	25
Adjuvant chemotherapy	8	9.6
Stage III	*n* = 21	100%
Chemoradiation for locally advanced disease	7	33.3
Stage IV	*n* = 62	100%
Metastatic sites at start of first palliative CHT		
- Liver	40	64.5
- Lung	15	24.2
- Peritoneum	14	22.6
- Bone	3	4.8
- Other	4	6.5
Number of metastatic sites		
- 1	50	80.6
- ≥ 2	12	19.4
CA 19.9 (U/l)		
- Median (range)	1363	(0–328,209)
- ≤ 37	14	16.9
- ≥ 37	69	83.1

Twenty-one patients had locally advanced disease and were treated either with FOLFIRINOX or nabPGem. This resulted in a similar OS (*p* = 0.35) when compared to that of the overall cohort. The very small subgroup of patients with locally advanced disease who received additional chemoradiation (*n* = 7) after induction with systemic chemotherapy had a significantly better outcome, with a median OS of 28.4 months (95% CI: 4.1–52.7, *p* = 0.044).

### Systemic chemotherapy: Efficacy

**Fig. 1:**
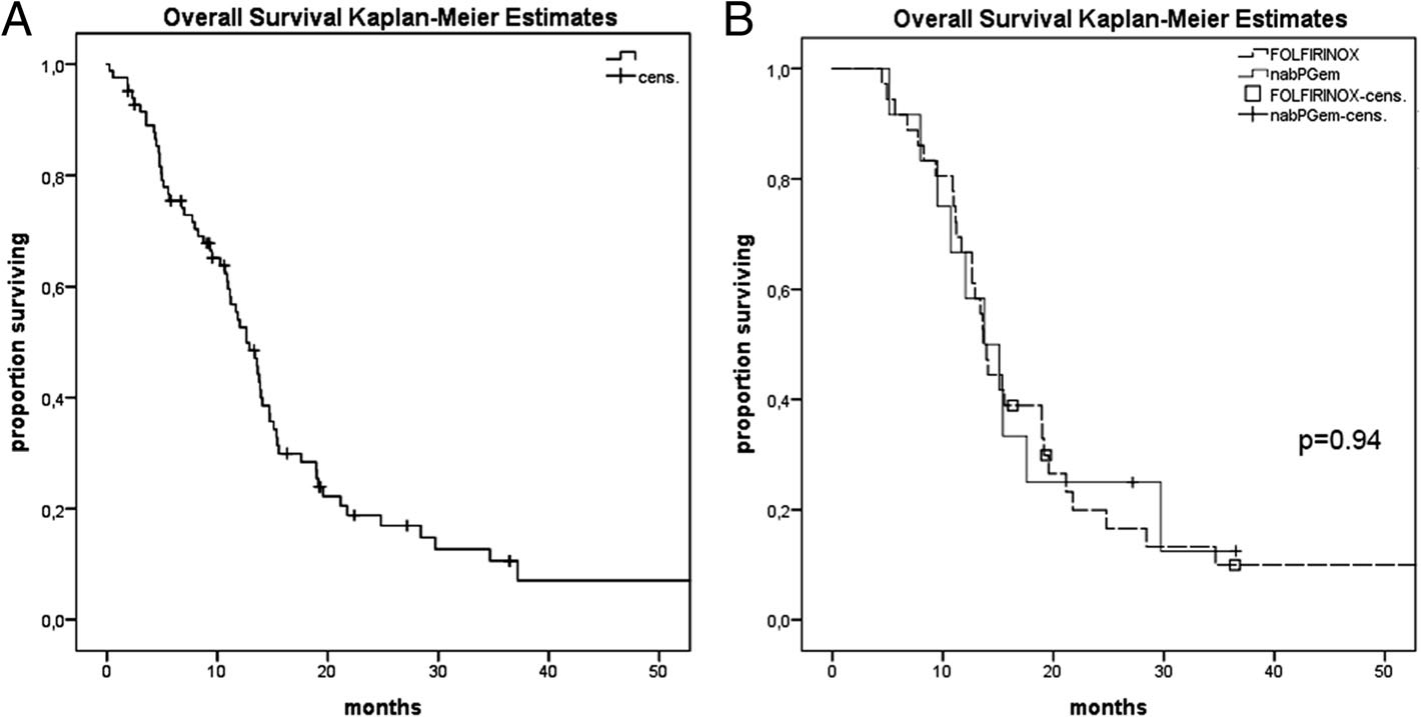
**a**: Overall survival (OS) of 91 metastatic or locally advanced patients measured from the first dose of palliative chemotherapy. Median OS: 13.4 months (95% CI: 11.6–15.2). **b**: Overall survival of patients receiving first-line FOLFIRINOX and second-line nabPGem (median OS: 13.65 months (95% CI: 12.6–14.7) vs. nabPGem followed by FOLFIRINOX (median OS: 13.78 months (95% CI: 8.6–19), *p* = 0.94

The median OS from the initiation of first palliative chemotherapy for locally advanced or metastatic pancreatic patients was 12.6 months (95% CI: 10.7–14.6, Fig. 1a). The one-year and two-year OS rates were 54 and 19%, respectively. ECOG PS (0 vs 2) was the only significant prognostic factor for OS (*p* < 0.001) in the univariate analysis.

The total number of treatment lines and chemotherapy regimens received are presented in Table 2 in detail. First-line palliative chemotherapy with FOLFIRINOX was administered to 51% of patients (*n* = 42), and 49% of patients (*n* = 41) received nabPGem up front. The median PFS of nabPGem and FOLFIRINOX as first-line therapy was 4.77 months (95% CI: 2.73–6.82) and 6.41 months (95% CI: 4.31–8.52), respectively. The ORR of FOLFIRINOX was 43, and 45% had stable disease (SD). First-line nabPGem led to an ORR of 20%, with a clinical benefit rate in two-thirds of patients (Table 3).

**Table 2:** Chemotherapy regimens received

	*N*	%
First-line	83	100
- FOLFIRINOX	42	50.6
- nabPGem	41	49.4
- Death after 1st-line or not	16	19.3
- suitable for 2nd-line		
- too early (1st-line ongoing)	3	3.6
Second-line	64	100
- FOLFIRINOX	13	20.3
- nabPGem	35	54.7
- Nal-IRI	11	17.2
- FOLFOX	2	3.1
- FOLFIRI	2	3.1
- Gem/Erlotinib	1	1.6
- Death after 2nd-line or not suitable for 3rd-line	19	29.7
- too early (2nd-line ongoing)	10	15.6
Third-line	35	100
- FOLFIRINOX re-induction	8	22.9
- nabPGem	6	17.1
- FOLFOX	2	5.7
- Gem/Erlotinib	11	31.4
- *nab*-Pacl/5-FU	1	2.9
- *nal*-IRI	5	14.3
- FOLFIRI	3	8.6
- *nal*-IRI/Erlotinib	2	5.7
> Fourth-line	17

**Table 3:** ORR of FOLFIRINOX and nabPGem in 1st and 2nd line

Efficacy parameter	FOLFIRINOX (*n* = 55)		nabPGem (*n* = 76)	
	1st-line	2nd-line	1st-line	2nd-line
	(*n* = 42)	(*n* = 13)	(*n* = 41)	(*n* = 35)
Response rate	*n* (%)	*n* (%)	*n* (%)	*n* (%)
PR	18 (43)	3 (23)	8 (20)	3 (9)
SD	19 (45)	7 (54)	22 (54)	19 (54)
PD	5 (12)	3 (23)	11 (27)	13 (37)

Second-line treatment was offered to 80.7% of eligible patients (*n* = 64). Three patients were still on first-line treatment. Treatment mostly consisted of nabPGem, FOLFIRINOX or nal-IRI. After failure of second-line treatment, 54% of patients (*n* = 35) were eligible to receive third-line treatment (10 patients still in second-line). Overall, the median number of treatment lines was three (range 1–7). Patients who received three or more treatment lines had a favourable OS of 14.1 months (95% CI: 12–16.1).

### Systemic chemotherapy: Sequence of FOLFIRINOX and nabPGem

**Fig. 2:**
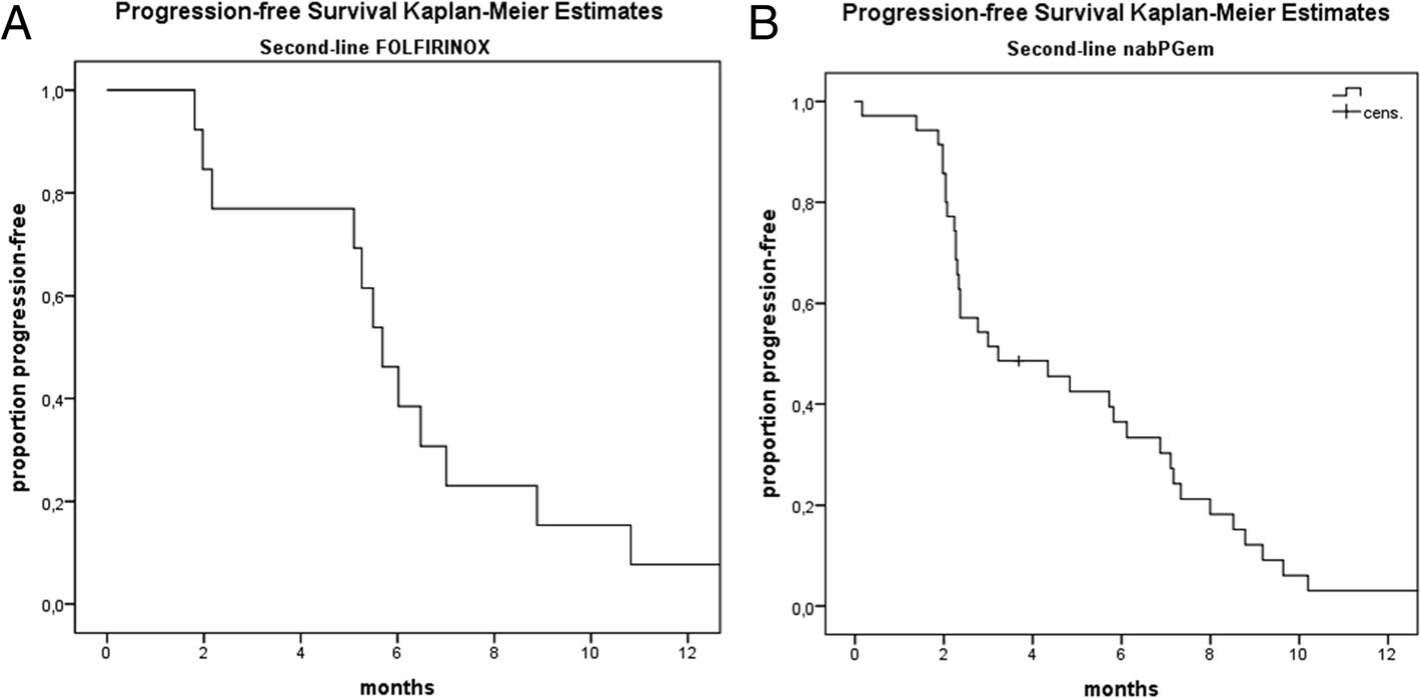
**a**: Progression-free survival of patients receiving nabPGem as second-line therapy after FOLFIRINOX pre-treatment: median PFS: 3.2 months (95% CI: 0.4–6). **b**: Progression-free survival of patients receiving FOLFIRINOX as second-line therapy after nabPGem pre-treatment: 5.7 months (95% CI: 4.8–6.6)

We analysed the sequence of nabPGem followed by FOLFIRINOX and FOLFIRINOX followed by nabPGem in 48 patients. Patient characteristics did not differ in the FOLFIRINOX or nabPGem first-line group concerning the number of metastases, age or stage, but ECOG PS was better in the FOLFIRINOX than in the nabPGem group (ECOG 0: 83% vs 63%, respectively). Nevertheless, patients with ECOG 1 in the nabPGem group were treated with FOLFIRINOX or mFOLFIRINOX as a second-line treatment. This difference in PS did not influence the OS outcome. There was no significant difference between the two sequences. Patients who received nabPGem followed by FOLFIRINOX had a median OS of 13.78 months (95% CI: 8.6–19) versus 13.65 months (95% CI: 12.6–14.7) for the patients receiving FOLFIRINOX followed by nabPGem (*p* = 0.94, Fig. 1b). The median PFS of nabPGem after progression on FOLFIRINOX was 3.2 months (95% CI: 0.4–6) and 5.7 months (95% CI: 4.8–6.6) second-line FOLFIRINOX after nabPGem (Fig. 2a, b).

### Systemic chemotherapy: Toxicity

mFOLFIRINOX (80% dose) was applied to 22% of patients, mostly as second-line treatment due to reduced ECOG or expected toxicity. The majority of patients (85%) needed dose reduction during the course of treatment due to neutropenia (61%) or polyneuropathy (26%). We performed de-escalation after FOLFIRINOX to 5-FU, leucovorin, and irinotecan (FOLFIRI) due to polyneuropathy (PNP) in 28% of patients. Adverse events of grade 3 and higher were experienced by 35% of patients. The most frequent side effects were neutropenia and PNP.

Patients receiving nabPGem had a lower prevalence of grade 3 PNP than patients in the FOLFIRINOX group, occurring in 14% of nabPGem treated patients. Grade 3 neutropenia (13%), thrombopenia (8%) and alopecia (81%) were the main other adverse events, leading to dose delay (30%), dose reduction or a change in treatment schedule to a biweekly application in 37% of patients. A select group of patients was treated on a biweekly schedule from baseline due to lower PS (68%). We did not observe a difference in OS between the standard and the biweekly schedule (*p* = 0.147).

## Discussion

Despite the implementation of modern cytotoxic agents in the treatment of metastatic pancreatic cancer, the reported one-year survival rate is less than 20%. Our data show a significantly higher 1-year OS rate (56%) than the published data. We suggest that two major treatment strategies led to this outstanding OS data in our patient cohort. First, we offered multiple chemotherapy lines with continuous, toxicity-adapted treatment whenever PS and patient preference allowed this strategy.

Second, the choice of first-line treatment, FOLFIRINOX or nabPGem, did not influence the decision to continue treatment with the alternative regimen in the second-line setting. Therefore, a considerable number of patients were able to receive and tolerate treatment with the two most effective chemotherapy regimens known to date.

Our patient cohort received a median of three treatment lines, which, in part, possibly improved outcomes in our patient cohort, as discussed earlier. In the literature, there are little data on patients receiving two or more treatment lines. One explanation for the high percentage of second-line treated patients at our institution could be a combination of careful observation for clinical deterioration, CA19–9 monitoring if applicable, and treatment until progression with a low rate of treatment interruption. Chemotherapy had to be adapted since toxicity, in particular PNP, occurred in almost 80% of patients during prolonged application of oxaliplatin. Due to PNP, the chemotherapy regimen of nearly one-third of the patients who showed an initial response to FOLFIRINOX was de-escalated to FOLFIRI. Early dose modifications or cessation of oxaliplatin is a key factor for maintaining quality of life. Treatment continuation with alternative drugs harbouring less toxicity is important. Given the patient’s compliance, tolerability and PS, FOLFIRI was administered until disease progression. We think that treatment interruptions in this aggressive disease are acceptable only under stringent medical controls, since progression often leads to a rapid deterioration in patient PS. CA19–9 seems to be a useful marker for monitoring patients during palliative treatment and during drug holidays. Together with clinical parameters and radiographic imaging, CA 19–9 may also help us identify patients who might benefit from an earlier switch to second-line treatment. Our treatment strategy with maintenance therapy was underlined at this year’s American Society of Clinical Oncology (ASCO) annual meeting with the phase II PRODIGE 35-PANOPTIMOX trial, which investigated FOLFIRINOX for 6 months or FOLFIRINOX for 4 months with 5-FU maintenance [25]. The median OS was longer in the maintenance arm with 5-FU. Interestingly, patients’ median duration of 5-FU was only 3.3 months, despite this being a usually well-tolerated chemotherapy. Our maintenance therapy mostly consisted of FOLFIRI until progression, which was also well tolerated and might be more effective than 5-FU monotherapy.

In the future, we will continue the approach of multiple treatment lines when choosing nabPGem as a first-line treatment, followed by mFOLFOXIRI or nal-IRI, as recommended in guidelines. A good third-line option might be 5-FU and folinic acid (FOLFOX) for patients following nal-IRI, even for patients with lower PS and resolving PNP.

The second question we addressed in our analysis was the choice and sequence of first- and second-line chemotherapy. In the literature, two effective first-line regimens have been shown to improve OS in this disease. For many years, FOLFIRINOX was the first-line treatment of choice for patients with a good PS. The percentage of patients with good PS in the real-world population is usually significantly lower, and patients with a poor PS (ECOG 2) are rarely included in clinical trials. We treated approximately half of our patients between 2012 and 2018 with first-line FOLFIRINOX. Until the approval of nabPGem [18], this scheme was the only combination with significant response rates. After the incorporation of nabPGem into guidelines, our patients were offered both options. Therefore, a comparison of the efficacy between first- and second-line choices was possible.

Treatment selection was mainly driven by patient preference, PS and availability. Patient preference for first-line regimen choice is a factor that has been included in the recent guidelines of the ASCO for pancreatic cancer [26]. Therefore, both options had to be discussed with individual patients in the eligible patient cohorts. Age and tumour burden did not differ significantly between these groups. Not surprisingly, patients with ECOG PS 0 were found more often in the FOLFIRINOX first-line group. Nevertheless, patients with impaired PS received FOLFIRINOX or mFOLFIRINOX in the following treatment lines. Therefore, ECOG PS was not a factor that influenced OS outcome when both lines were applied, highlighting the importance of continuous treatment adapted to every patient’s individual needs by offering dose modifications.

There is limited evidence in the literature regarding whether the choice of first-line treatment and sequence is important for outcome in patients with pancreatic cancer. However, there are reports, mostly case studies, summarizing the experience of second-line nabPGem after initial treatment with FOLFIRINOX [27–31]. The largest report is from Portal and colleagues, who enrolled 57 patients [32]. Moreover, there are abundant data regarding these two regimens administered as first-line treatment, in which treatment duration, efficacy as measured by response and costs [21, 33–35] are reported. A recent chart review from different US centers has been published, reporting over 600 patients receiving either FOLFIRINOX or nabPGem as first-line chemotherapy [20]. FOFIRINOX and nabPGem were equally effective as first-line treatment as reported in this publication. Only about a third of patients received a second-line treatment with a great variety of protocols.In contrast, we present unique data with a smaller, yet more homogenous patient cohort, with sequence data of patients who received both treatments (FOLFIRINOX and nabPGem) as subsequent protocols.

Our patient series and experience over the past few years were used to address and answer – although in a small patient cohort – the lack of data regarding these factors, namely, sequence and efficacy of second and further lines of chemotherapy. We showed that the sequence of these two regimens did not influence OS, and both groups had a median survival of approximately 14 months. Moreover, our report also indicates that second-line treatment after nabPGem with FOLFIRINOX is possible and effective in a considerable number of patients, even when dose reductions and modifications of the original protocol were necessary in patients with a reduced PS.

The subgroup of patients with locally advanced disease at our institution has been treated with the intention of long-term local control and prevention of metastatic spread. European and American guidelines differ significantly in the treatment of locally advanced disease. The ESMO guidelines recommend gemcitabine monotherapy for this patient population [36]. NCCN guidelines [22] recommend systemic chemotherapy (4–6 months) similar to the treatment for metastatic patients, followed by chemoradiation for selected patients. These recommendations for inoperable patients with locally advanced disease were derived from extrapolations from randomized trials in patients with metastatic disease. Prospective data for these patients are lacking. Our patient cohort included a considerable number of those with locally advanced disease (24%). We treated most of these patients with a good PS with induction chemotherapy with FOLFIRINOX. One-third of all patients with localized disease also received chemoradiation after a period of 3 to 6 months. The outcome of locally advanced disease and metastatic disease did not differ in our patient cohort. We showed – although in a very small patient subgroup – that patients receiving systemic chemotherapy and local radiotherapy experience an impressive median OS of more than two years. Recent data from the ASCO 2018 meeting (PREOPANC-1 trial) suggest a survival benefit with neoadjuvant chemoradiation [37].

## Conclusions

In summary, our data showed that the sequence of use of FOLFIRINOX and nabPGem did not influence the outcome. FOLFIRINOX is effective and tolerable as a second-line treatment after nabPGem when adapted to patients’ individual needs, such as PS and underlying toxicities from first-line treatment. Our approach of adapted treatment and maintenance led to an impressive OS outcome. To the best of our knowledge, this is the largest comparative report of patients receiving both recommended treatments.

## Abbreviations

5-FU: 5-Fluouracil
BRCA: Breast cancer type 1 susceptibility protein
CA19.9: Carbohydrate Antigen 19.9
CTCAE: Common Terminology Criteria for Adverse Events
dMMR: deficient mismatch repair
ECOG: European Cooperative Oncology Group
ESMO: European Society of Medical Oncology
FOLFIRI: 5-FU, Leukovorin, Irinotecan
FOLFIRINOX: 5-FU, Leukovorin, Irinotecan, Oxaliplatin
mFOLFIRINOX: modified 5-FU, Leukovorin, Irinotecan, Oxaliplatin
MSI: Microsatellite instability
nabPGem: nab-Paclitaxel/Gemcitabine
NCCN: National Comprehensive Cancer Network
OS: Overall Survival
PFS: Progression-free survival
PNP: Polyneuropathy
PS: Performance status
RAS: Rat sarcoma
RECIST: Response Evaluation Criteria in Solid Tumors
TP53: Tumor protein 53
